# Fetal Adrenal Demedullation Lowers Circulating Norepinephrine and Attenuates Growth Restriction but not Reduction of Endocrine Cell Mass in an Ovine Model of Intrauterine Growth Restriction

**DOI:** 10.3390/nu7010500

**Published:** 2015-01-09

**Authors:** Melissa A. Davis, Antoni R. Macko, Leah V. Steyn, Miranda J. Anderson, Sean W. Limesand

**Affiliations:** School of Animal and Comparative Biomedical Sciences, The University of Arizona, Tucson, AZ 85721, USA; E-Mails: melissaad@email.arizona.edu (M.A.D.); tonymacko@gmail.com (A.R.M.); penrodl@email.arizona.edu (L.V.S.); miranda1@email.arizona.edu (M.J.A.)

**Keywords:** catecholamines, fetal growth, insulin, pregnancy

## Abstract

Placental insufficiency is associated with fetal hypoglycemia, hypoxemia, and elevated plasma norepinephrine (NE) that become increasingly pronounced throughout the third trimester and contribute to intrauterine growth restriction (IUGR). This study evaluated the effect of fetal adrenal demedullation (AD) on growth and pancreatic endocrine cell mass. Placental insufficiency-induced IUGR was created by exposing pregnant ewes to elevated ambient temperatures during mid-gestation. Treatment groups consisted of control and IUGR fetuses with either surgical sham or AD at 98 days gestational age (dGA; term = 147 dGA), a time-point that precedes IUGR. Samples were collected at 134 dGA. IUGR-sham fetuses were hypoxemic, hypoglycemic, and hypoinsulinemic, and values were similar in IUGR-AD fetuses. Plasma NE concentrations were ~5-fold greater in IUGR-sham compared to control-sham, control-AD, and IUGR-AD fetuses. IUGR-sham and IUGR-AD fetuses weighed less than controls. Compared to IUGR-sham fetuses, IUGR-AD fetuses weighed more and asymmetrical organ growth was absent. Pancreatic β-cell mass and α-cell mass were lower in both IUGR-sham and IUGR-AD fetuses compared to controls, however, pancreatic endocrine cell mass relative to fetal mass was lower in IUGR-AD fetuses. These findings indicate that NE, independently of hypoxemia, hypoglycemia and hypoinsulinemia, influence growth and asymmetry of growth but not pancreatic endocrine cell mass in IUGR fetuses.

## 1. Introduction

Intrauterine growth restriction (IUGR) is often caused by placental insufficiency resulting in fetal hypoxemia and hypoglycemia, which elevate plasma catecholamines [1,2,3,4,5,6,7,8,9,10]. Elevated plasma norepinephrine (NE) concentrations have been reported in human IUGR fetuses and in animal models of placental insufficiency-induced IUGR [3,11,12,13,14,15]. In fetal sheep, NE is the major catecholamine secreted from the adrenal medulla due to low expression of phenylethanolamine N-methyltransferase (PNMT), an enzyme that converts NE to epinephrine [16]. Although sympathetic neurons, such as the splanchnic nerve, contribute to plasma NE concentrations, the adrenal medulla has been shown to be primarily responsible for hypoxia-stimulated increases of NE and epinephrine in fetal plasma [13,17,18,19,20]. High NE concentrations inhibit fetal insulin secretion via α_2_-adrenergic receptors on pancreatic β-cells [3,12,13,14,15,17,19,21,22,23,24,25,26,27,28,29]. Expression of α_2A_-adrenergic receptor mRNA was greater in islets isolated from the IUGR fetus, which potentially facilitate the chronic inhibition of insulin, an anabolic hormone in the fetus [3,4,5,30,31]. Previous work has shown that continuous infusion of epinephrine or NE for >7 days into normal sheep fetuses produced asymmetric fetal growth restriction and chronically lowered plasma insulin concentrations [17,32]. In contrast to IUGR islets, the α_2A_-adrenergic receptor mRNA concentrations were lower in NE-infused islets [32]. Pancreatic endocrine cell mass was unaffected by NE-infusion, whereas in IUGR fetuses β-cell mass was substantially lower because of slower β-cell replication rates [4,32,33]. Exogenous replacement of insulin during the NE infusion counteracted the growth restriction, which indicates that low insulin concentrations were essential for decreased growth rates in fetuses with normal oxygen and glucose concentrations [30,32]. Additionally, insulin-independent NE actions have been associated with lower oxidative metabolism [20,30]. Together, these findings show that chronically elevated plasma NE concentrations restrict fetal growth, promote asymmetric growth, and suppress insulin concentrations in otherwise normal fetuses, however NE actions have not been defined in IUGR fetuses with low blood glucose and oxygen concentrations.

In IUGR fetuses, metabolic and endocrine modifications promote glucose and oxygen sparing to preserve necessary fetal functions but also result in stunted and asymmetrical fetal growth. However, the contribution of adrenal catecholamine secretion to alter growth trajectory has not been evaluated [5,34,35]. Therefore, the objective of this study was to determine the specific effects of chronically elevated NE, independent of hypoxemia and hypoglycemia, on fetal growth and pancreatic endocrine cell composition in the near-term, PI-IUGR sheep fetus. This was accomplished by surgical ablation of the fetal adrenal medulla and therefore, fetal NE responsiveness to hypoxemia and hypoglycemia. The bilateral adrenal demedullation was performed at 98 days gestational age (dGA; term = 147 dGA), a time point our group has previously determined to precede the onset of IUGR and is predicted to preceded the elevation in fetal plasma NE in this model of placental insufficiency-induced IUGR [30,33,36,37]. Fetal measurements were collected at 134 dGA, a time point at which fetal mass was 50% lower in IUGR fetuses compared to controls [36,37].

## 2. Experimental Section

### 2.1. Ethical Approval

Columbia-Rambouillet crossbred ewes (62 ± 3 kg) carrying singleton pregnancies were purchased from Nebeker Ranch (Lanscaster, CA, USA). Ewes were fed Standard-Bread Alfalfa Pellets (Sacate Pellet Mills) and provided water *ad libitum*. Protocols were conducted with approval by the Institutional Animal Care and Use Committee at The University of Arizona Agricultural Research Complex, Tucson, AZ, USA.

### 2.2. IUGR and Control Fetuses

Singleton pregnancy was confirmed by ultrasound at approximately 35 days gestation age. Ewes were randomly assigned to either a control (*n* = 15) or IUGR (*n* = 11) treatment. Placental insufficiency-IUGR was created by exposing pregnant ewes to elevated ambient temperatures (40 °C for 12 h; 35 °C for 12 h; dew point 22 °C) from 39 ± 1 dGA until 94 ± 1 dGA (0.27–0.63 gestation) as described previously [4]. Control fetuses were from healthy pregnant ewes that were maintained at 25 °C and pair-fed to the average daily feed intake of IUGR ewes. Feed was withheld 24 h prior to surgery.

### 2.3. Surgical Preparation for Adrenal Demedullation and Cannulation

At 98 ± 1 dGA control and IUGR fetuses were randomly assigned to undergo either a bilateral adrenal demedullation (AD) or sham (placebo) surgical procedure. Fetal adrenal glands were isolated via retroperitoneal incisions and a straight electrode was used to cauterize the inner medullary tissue while leaving the cortex intact as previously described [20]. At 121 ± 1 dGA, each fetus underwent a second surgical procedure to place indwelling, polyvinyl arterial and venous catheters for blood sampling and infusion as described previously [5,38]. Animals were allowed to recover for at least one week prior to fetal blood collection experimental procedures. The catheters were flushed daily with heparinized saline solution (100 U/mL heparin in 0.9% NaCl solution, Vedco, Inc, St. Joseph, MO, USA). The final treatment designations were control-sham (*n* = 8), control-AD (*n* = 7), IUGR-sham (*n* = 7), and IUGR-AD (*n* = 4) fetuses.

### 2.4. Postmortem Examination

The ewe and fetus (134 ± 1 dGA) were euthanized with intravenous concentrated pentobarbital sodium (86 mg/kg) and phenytoin sodium (11 mg/kg, Euthasol; Virbac Animal Health, Fort Worth, TX, USA). After a hysterectomy, the fetus was removed, blotted dry, and weighed. Fetal organs were dissected and weighed. The fetal pancreas was dissected free, weighed, and divided from the common bile duct to the anatomic left side of the portal vein (pancreatic notch, when visible). Pancreas masses were obtained from control-sham (*n* = 7), control-AD (*n* = 5), IUGR-sham (*n* = 4), and IUGR-AD (*n* = 3) fetuses due to other procedures.

### 2.5. Biochemical Analysis

Arterial blood gases were measured with an ABL 720 (Radiometer, Copenhagen, Denmark) and values were temperature-corrected to 39.1 °C, the average core body temperature for sheep. Plasma glucose concentrations were quantified with the YSI model 2700 SELECT Biochemistry Analyzer (Yellow Springs Instruments, Yellow Springs, OH, USA). Plasma concentrations of insulin (ovine insulin ELISA; ALPCO Diagnostics, Windham, NH; intra- and inter-assay coefficients of variation <6%; sensitivity 0.14 ng/mL), norepinephrine (noradrenaline ELISA; Labor Diagnostika Nord GmbH & Co., KG, Germany; intra- and inter-assay coefficients of variation <14%; sensitivity 35 pg/mL), and cortisol (ALPCO Diagnostics; intra- and inter-assay coefficients for variation <10%; sensitivity 2.44 ng/mL) were quantified.

### 2.6. Pancreas Morphology and Pancreatic Endocrine Cell Proliferation

The splenic portion of the fetal pancreas was collected at necropsy and fixed in 4% paraformaldehyde and embedded in O.C.T Compound (Sakura Finetek USA, Torrance, CA, USA) as previously described [4,39]. Pancreas sections were cut and analyzed at 100 μm intervals. Immunofluorescent staining identified mature cell types within the pancreas: β-cells with guinea pig anti-porcine insulin (1:500; Dako, Carpinteria, CA, USA); α-cells with mouse anti-porcine glucagon (1:250; Sigma-Alrich, St. Louis, MO, USA); δ-cells with rabbit anti-human somatostatin (1:500; Dako, Carpinteria, CA, USA); F cells with rabbit anti-human pancreatic polypeptide (PP) (1:500; Dako, Carpinteria, CA, USA). β-cells in mitosis were identified with rabbit polyclonal phosphorylated-Histone H3 (pHH3; 7.5 µg/mL; Upstate, Lake Placid, NY, USA), anti-insulin, and DAPI (4′,6-diamidino-2-phenylindole; 1 mg/mL; Vector Laboratories, Burlingame, CA, USA). Secondary antiserum against the appropriate species immunoglobulin conjugated to 7-amino-4-methylcoumarin-3-acetic acid (AMCA; Jackson ImmunoResearch Laboratories, West Grove, PA, USA), AlexaFluor 488 (Molecular Probes, Eugene, OR, USA), or AlexaFluor 594 (Molecular Probes, Eugene, OR, USA) were used for detection. The fluorescent images were visualized on the Leica DM5500 microscope system and digitally captured with a Spot Pursuit 4 Megapixel CCD camera (Diagnostic Instruments, Sterling Heights, MI, USA). Morphometric analysis was performed with ImagePro 6.3 software (Media Cybernetics, Silver Spring, MD, USA). Positive areas were determined for 25 fields of view on at least two pancreas sections per animal separated by ≥100-μm intervals. Data are expressed as a percentage of total pancreas area. Phosphorylated-Histone H3 positive cells were determined per 3000–4000 DAPI positive cells within β-cells on at least two pancreas sections per animal separated by ≥100-μm intervals. Data are expressed as a percentage of the total number of DAPI positive cells per insulin positive cell. β-cell mass (mg per pancreas) was calculated by multiplying relative insulin-positive area (the percentage of insulin positive area over total pancreas area) by pancreas mass.

### 2.7. Statistical Analysis

Significant differences (*p* < 0.05) among treatments were determined with an ANOVA and post hoc LSD test using general linear means procedures in the Statistical Analysis System (SAS Institute, Cary, NC, USA, v9.2, 2008). Data are presented as mean ± SEM.

## 3. Results

### 3.1. Fetal Biometry and Blood Measurements

**Figure 1:**
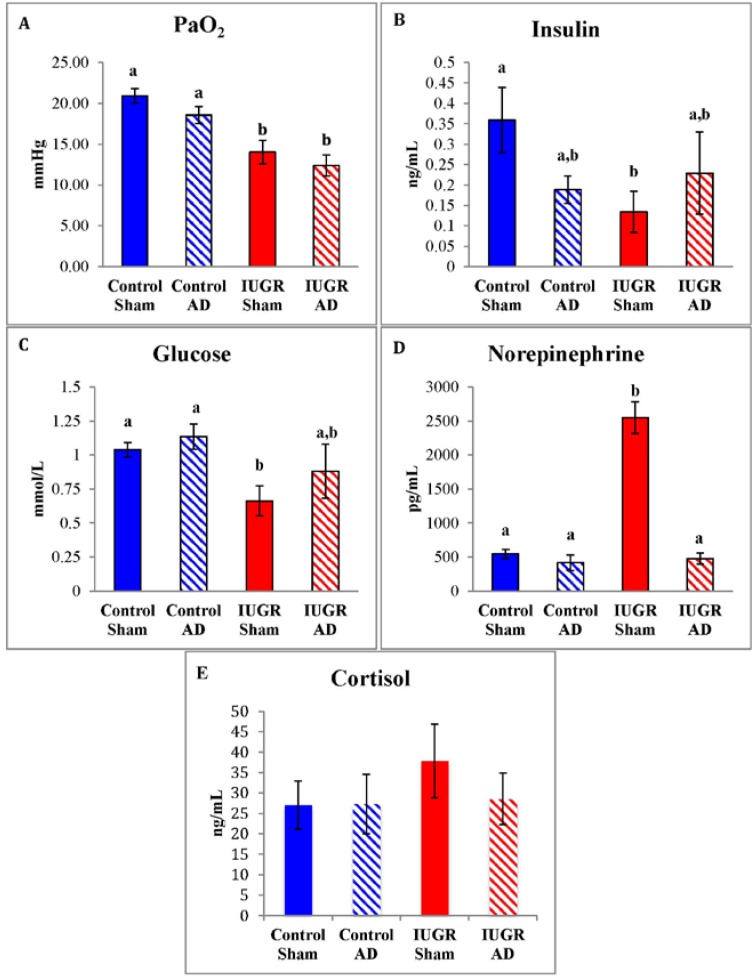
Fetal arterial oxygen tension and plasma glucose and hormone concentrations. Means (±SEM) for arterial blood oxygen tension (PaO_2_; **A**); insulin (**B**); glucose (**C**); norepinephrine (**D**); and cortisol (**E**) are graphed for control-sham, control-adrenal demedullation (AD), intrauterine growth restriction (IUGR)-sham, and IUGR-AD treatment groups (x-axis). Error bars represent the SEM and different superscripts denote statistically significant differences among treatment groups (*p* < 0.05).

Arterial blood oxygen tension was lower in IUGR-sham and IUGR-AD fetuses compared to control-shams and control-AD fetuses (Figure 1A). IUGR-sham fetuses had lower plasma insulin and glucose concentrations compared to control-shams (Figure 1). Fetal plasma NE concentrations were five-fold higher in the IUGR-sham fetuses compared to IUGR-AD fetuses, as well as control-sham and control-AD fetuses (Figure 1D). Plasma NE concentrations were not different among IUGR-AD fetuses and the control groups. There were no differences in plasma cortisol concentrations among treatment groups. (Figure 1E).

There were no differences in overall mass or the masses of individual organs between control-sham and control-AD fetuses (Table 1). Compared to the control groups, the mean fetal mass was 51% lower in the IUGR-sham group and 26% lower in the IUGR-AD group. Placenta mass was lower in the IUGR groups compared to the control groups, but were not different among the IUGR and control treatments. Placental efficiency, calculated as the ratio of fetal to placental mass, was greater in IUGR-AD fetuses (15.4 ± 0.9 g) compared to IUGR-sham (11.8 ± 0.7 g), control-sham (10.7 ± 0.9 g), and control-AD (11.4 ± 0.7 g) fetuses. Compared to other treatment groups, neural tissue (cerebrum, cerebellum, and brain stem) mass was lower in the IUGR-shams. Heart and kidney masses were lower in IUGR-shams as well as IUGR-AD fetuses compared to both control groups. Liver mass was greater in the control-sham group compared to both IUGR groups. Pancreatic mass for control-sham (*n* = 7), control-AD (*n* = 5), IUGR-sham (*n* = 4), and IUGR-AD (*n* = 3) was 3.43 ± 0.23 g, 2.92 ± 0.22 g, 2.38 ± 0.48 g, 2.73 ± 0.50 g, respectively. In IUGR-shams, pancreatic mass was lower compared to both control groups and the IUGR-AD group.

**Table 1:** Mean (±SEM) fetal, placental, and organ masses (g) for treatment groups.

Treatment Group	Control Sham (*n* = 8)	Control AD (*n* = 7)	IUGR Sham (*n* = 7)	IUGR AD (*n* = 4)
**Fetus**	3250 ± 149 ^a^	3242 ± 177 ^a^	1577 ± 250 ^b^	2394 ± 414 ^c^
**Placenta**	319 ± 27 ^a^	272 ± 8.7 ^a^	137 ± 26 ^b^	160 ± 36 ^b^
**Brain**	50.5 ± 1.4 ^a^	50.0 ± 1.7 ^a^	40.9 ± 2.1 ^b^	48.8 ± 3.1 ^a^
**Heart**	22.6 ± 1.2 ^a^	22.3 ± 1.6 ^a^	12.2 ± 1.8 ^b^	16.6 ± 1.9 ^b^
**Kidneys**	22.4 ± 1.1 ^a^	23.3 ± 1.3 ^a^	11.7 ± 2.0 ^b^	17.3 ± 1.1 ^c^
**Liver**	96.5 ± 8.5 ^a^	88.3 ± 5.2 ^a,b^	45.0 ± 7.8 ^c^	63.5 ± 11 ^b,c^

Treatments with different superscripts differ (*p* < 0.05).

**Figure 2:**
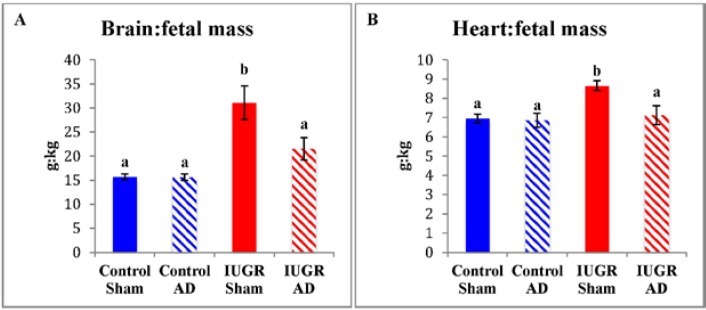
Brain and heart mass relative to fetal mass. Mean brain mass (**A**) and heart mass (**B**) relative to fetal mass (g/kg) are presented for the treatment groups labeled on the x-axis. The treatment groups are presented on the x-axis. Error bars represent the SEM and different superscripts denote statistically significant differences among treatment groups (*p* < 0.05).

The mass of fetal organs in proportion to fetal mass (g/kg) revealed sparing of brain and heart in IUGR-sham fetuses compared to all other treatment groups (Figure 2). In IUGR-AD fetuses, the relative brain and heart proportions were not different from either control group. There were no differences in the relative mass of liver (control-sham, 29.4 ± 1.8 g; control-AD, 27.4 ± 1.4 g; IUGR-sham, 31.5 ± 2.0 g; IUGR-AD, 26.6 ± 1.6 g) and kidneys (control-sham, 6.9 ± 0.2 g; control-AD, 7.2 ± 0.4 g; IUGR-sham, 8.5 ± 1.3 g; IUGR-AD, 7.6 ± 0.7 g) between treatment groups. Relative pancreatic mass for IUGR-sham (1.41 ± 0.07 g/kg; *n* = 4) was greater than control-sham (1.03 ± 0.04 g/kg; *n* = 7), control-AD (0.97 ± 0.11 g/kg; *n* = 5), and IUGR-AD (1.09 ± 0.10 g/kg; *n* = 3).

### 3.2. Fetal Pancreas Morphology

**Figure 3:**
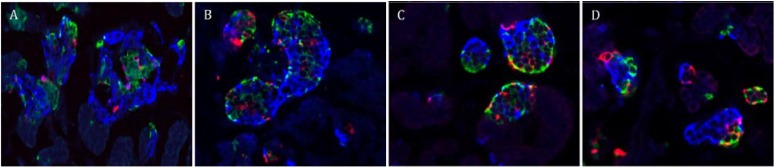
Immunostaining for pancreatic endocrine cells. Representative micrographs for pancreatic endocrine cells are depicted for control-sham (**A**); control-AD (**B**); IUGR-sham (**C**); and IUGR-AD (**D**) fetuses. The pancreatic sections were immunostained for insulin (β-cells; blue), glucagon (α-cell; green) and the combination of somatostatin and PP (δ-cells and F-cells; red).

**Figure 4:**
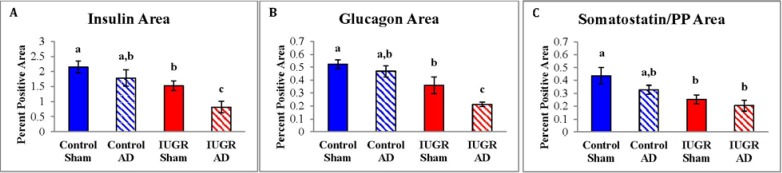
Pancreatic endocrine cell areas. Insulin positive area (**A**); glucagon positive area (**B**); and the combined somatostatin and PP positive area (**C**) were measured and the mean percent area of the pancreas tissue graphed for the treatment groups labeled on the x-axis. Error bars represent the SEM and different superscripts denote statistically significant differences among treatment groups (*p* < 0.05).

Mature pancreatic endocrine cells areas for control-sham, control-AD, IUGR-sham, and IUGR-AD fetuses were determined with immunofluorescent staining (Figure 3). In IUGR-AD fetuses, insulin (β-cell) and glucagon (α-cell) positive areas were lower than other treatment groups, although IUGR-sham fetuses were also lower compared to control-shams (Figure 4). The combined somatostatin and PP (δ- and F-cells) positive area was lower in IUGR-sham and IUGR-AD fetuses compared to control-sham fetuses (Figure 4C).

Endocrine cell masses were calculated as the product of percent area and pancreatic mass (Table 2). β-cell mass was lower in IUGR-sham and IUGR-AD fetuses compared to control-sham fetuses. β-cell mass in control-AD was not different from other treatment groups. Compared to control-sham fetuses, α-cell mass was lower in all other treatment groups. No differences in the combined somatostatin and PP mass were identified among treatment groups. The sum of the endocrine cell mass (total endocrine cell mass) was lower in IUGR-sham and IUGR-AD fetuses compared to control-sham fetuses. The total pancreatic endocrine cell mass also was lower in control-AD fetuses than control-sham fetuses.

**Figure 5:**
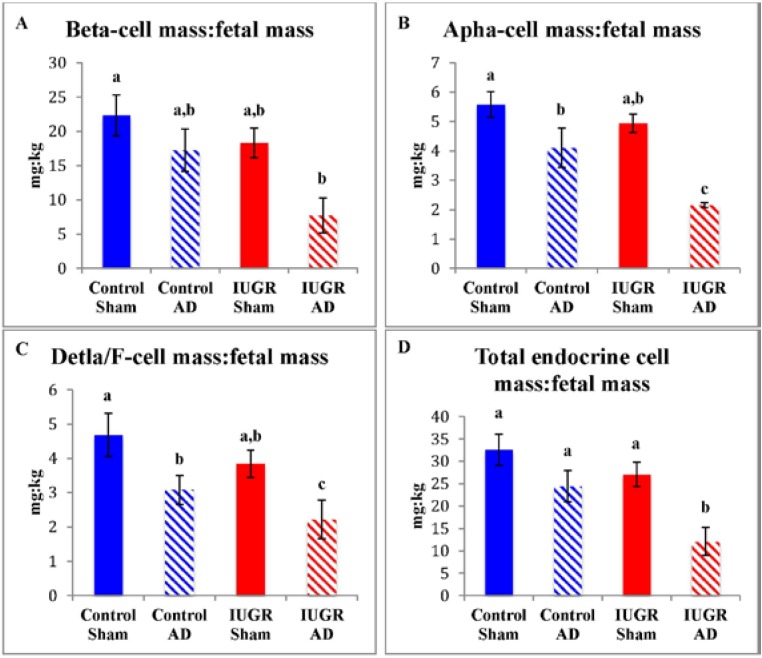
Endocrine cell mass relative to fetal mass. Means are graphed for β-cell (**A**); α-cell (**B**) combined δ-cell and F-cell (**C**); and total endocrine cell (**D**) mass expressed as a proportion of fetal mass (mg/kg). The treatment groups are presented on the x-axis. Error bars represent the SEM and different superscripts denote statistically significant differences among treatment groups (*p* < 0.05).

Total endocrine cell mass relative to fetal mass (mg/kg) was lower in IUGR-AD fetuses compared to all other treatment groups (Figure 5D). The relative β-cell mass for IUGR-AD fetuses was lower than control-shams and tended to be less than control-AD (*p* < 0.07) and IUGR-sham (*p* < 0.06) fetuses (Figure 5A). IUGR-AD fetuses had a lower relative α-cell mass and combined δ- and F-cell mass compared to all other treatment groups. In control-AD fetuses, relative α-cell mass and combined δ- and F-cell mass were lower compared to control-sham fetuses.

**Figure 6:**
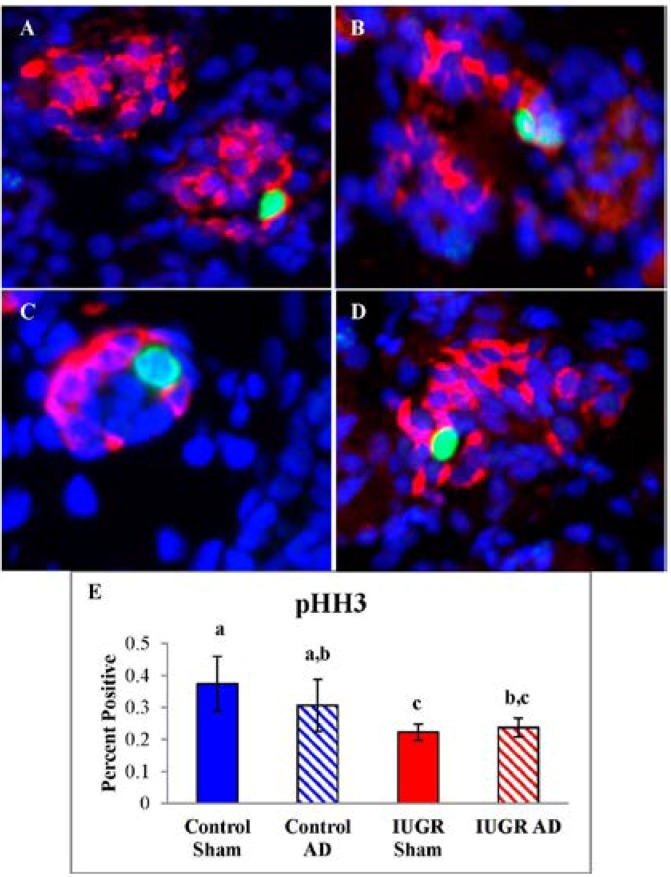
β-cell Proliferation Rates. Pancreas tissues were immunostained for phosphorylated-Histone H3 (pHH3; green), insulin (β-cells; red), and nuclear DNA (DAPI; blue). Representative micrographs are presented for control-sham (**A**); control-AD (**B**); IUGR-sham (**C**); and IUGR-AD (**D**). The mean (±SEM) percentages of pHH3 positive β-cells are graphed for treatment groups labeled on the x-axis (**E**). Error bars represent the SEM and different superscripts denote statistically significant differences among treatment groups (*p* < 0.05).

The proportion of pHH3 labeled β-cells was lower in IUGR-sham and IUGR-AD fetuses compared to control-shams (Figure 6E). No differences in pHH3 labeled β-cells were identified between control-sham and control-AD fetuses or between IUGR-sham and IUGR-AD fetuses.

**Table 2:** Mean (±SEM) endocrine cell mass (mg) for treatment groups.

Treatment Group	Control Sham (n = 7)	Control AD (*n* = 5)	IUGR Sham (*n* = 4)	IUGR AD (*n* = 3)
**β-cell Mass**	75.2 ± 12 ^a^	51.6 ± 8.4 ^a,b^	30.9 ± 7.8 ^b^	22.2 ± 12 ^b^
**α-cell Mass**	18.8 ± 2.1 ^a^	12.2 ± 1.6 ^b^	8.6 ± 2.2 ^b^	5.5 ± 1.4 ^c^
**δ- and F-cell Mass**	15.7 ± 2.2 ^a^	9.2 ± 1.1 ^a^	7.0 ± 2.2 ^a^	6.1 ± 2.7 ^a^
**Total Endocrine Cell Mass**	109.7 ± 14 ^a^	73.0 ± 8.7 ^b^	46.5 ± 12 ^b^	33.8 ± 16 ^b^

Treatment differences (*p* < 0.05) are denoted with different superscripts.

## 4. Discussion

Results of the experiments reported here provide strong support for the hypothesis that catecholamines secreted by the adrenal medulla, independently of hypoglycemia and hypoxemia, suppress the rate of growth and facilitate asymmetric organ growth in fetuses with placental insufficiency-induced IUGR. Previous studies have shown similar effects on fetal growth when plasma epinephrine and NE are increased chronically [17]. However, those experiments did not address the effects of catecholamines in the context of restricted fetal oxygen and nutrient supply imposed by placental insufficiency [10,40,41,42,43]. In the current study, ablation of the fetal adrenal medulla at the end of the second trimester of gestation prevented the rise in plasma NE concentrations, despite hypoxemic and hypoglycemic conditions, in near-term in fetuses with placental insufficiency-induced IUGR. Suppression of elevated plasma NE concentration, an indicator for adrenal catecholamine secretion, significantly improved the magnitude of fetal growth by ~50% in IUGR fetuses and curtailed asymmetric growth of brain and heart [20]. In IUGR-AD fetuses, reduction in pancreatic endocrine cell mass was unaffected by adrenal demedullation while the amount of endocrine mass relative to fetal mass was lower. These findings indicate that adrenal release of catecholamines in response to fetal hypoxemia and hypoglycemia secretion elevated NE concentrations and acted to inhibit somatic cell growth in IUGR fetuses; however, the mechanism appears to be independent of insulin because insulin concentrations remained low and pancreatic islet mass was unaffected.

Greater fetal growth restriction in intact IUGR fetuses indicates that the adrenal medulla plays a major role in coordinating inhibition of fetal growth and asymmetry of growth as glucose, oxygen, and insulin concentrations are similar among IUGR groups. Proposed mechanisms for the greater severity of IUGR with high catecholamines are redistribution of fetal blood flow, secondary actions on the regulation of anabolic hormones, and adaptations in metabolic substrate uptake and utilization in tissues. It has been shown that during acute hypoxia (<48 h), fetal blood flow increases to neural tissues, heart, and adrenals to maintain arterial oxygen delivery, whereas blood flow to kidneys, pancreas, and carcass remains constant or tends to decline [44,45,46,47,48]. The compensatory increases in blood flow to the brain and heart are not found in IUGR models with longer durations of hypoxemia but reduced blood flow to the carcass remains [49,50,51]. This may suggest changes in extraction efficiencies of tissues when catecholamines are chronically elevated, which is expected in neural tissues because glucose transporter 1 isoform concentrations are increased [5]. Furthermore, raising NE concentrations to values similar to those observed during hypoxemia also increased coronary blood flow, but the redistribution of blood flow was not identical to that observed in hypoxic fetuses [48]. Therefore, it has been postulated that altered endocrine regulation and substrate utilization are also potential mechanisms underlying IUGR. For example, NE or epinephrine infusions have been shown to increase insulin-like growth factor binding protein-1, which was associated with lowering insulin-like growth factor (IGF) bioavailability and DNA synthesis in select tissues [52,53,54]. Infusion of NE increases oxygen consumption in the fetus even though transplacental uptake of two major substrates for oxidative metabolism, lactate and amino acids, are reduced [55,56]. The net result is insufficient exogenous substrates for oxidative metabolism. Although not experimentally tested, this could partly explain reduced amino acid uptake from the placenta in IUGR fetuses with placental insufficiency [41,57]. Furthermore, fetal amino acid supplementation increased rates of fetal protein accretion in IUGR fetus rather than being utilized as oxidative substrates as observed in control fetuses [41]. Greater placental efficiency in IUGR-AD fetuses could be explained by the lack of NE regulation of fetal metabolism and placental uptake of amino acids.

Elevated plasma NE has been shown to acutely and chronically suppress plasma insulin concentrations, and adrenal demedullation impairs β-cell function [4,5,6,8,24,25,32,33]. In a previous study conducted in normal, near-term fetal sheep, ablation of adrenal medullae did not influence plasma glucose and insulin concentrations at baseline but lowered glucose-stimulated insulin concentrations, indicating that an intact adrenal medulla potentiates glucose-stimulated insulin concentrations [36]. Acute pharmacological antagonism of adrenergic receptors in IUGR fetuses improved glucose-stimulated insulin secretion at 103 dGA and 134 dGA, even though fetal hypoxemia was present [3,30,33]. Cessation of a 7-day NE infusion in normal sheep fetuses resulted in greater basal and glucose-stimulated insulin concentrations, which indicates chronically elevated NE enhanced post-treatment β-cell responsiveness [32]. In the present study, ablation of the fetal adrenal medullae at 98 dGA decreased the NE responsiveness to hypoglycemia and hypoxemia at 134 dGA, but plasma insulin concentrations remained low because the prior conditions of placental insufficiency (Figure 1). These findings are similar to results from chronically hypoglycemic sheep fetuses that have blunted insulin secretion responsiveness [38,58]. Therefore, NE or other hormones from the adrenal medulla regulate β-cell function under normal conditions but also enhance insulin secretion following chronic exposure to high NE concentrations.

Despite a greater fetal mass in IUGR fetuses with low NE, β-cell mass was similar to IUGR fetuses with high NE and in both IUGR groups β-cell mass was lower than control-sham fetuses. This indicates that IUGR conditions, not high NE, are responsible for reducing β-cell mass. This is consistent with the similar reduction in β-cell proliferation rate (Figure 6) and also consistent with other models in which fetal NE concentrations were elevated [32,59]. Several growth factors regulate β-cell proliferation and pancreas endocrine cell development [11,60,61,62]. IGF-I, fibroblast growth factor-7 (FGF), and FGF receptor 2IIIb mRNA concentrations are lower in the pancreas of the IUGR fetus and are proposed to decrease pancreatic progenitor epithelial cell expansion and subsequently reduces β-cell mass [33,63]. Insulin-like growth factor binding protein-2 (IGFBP-2) mRNA and protein concentrations are greater the fetal pancreas and islets of IUGR fetuses, which is postulated to antagonize IGF actions in β-cells [63]. Hepatocyte growth factor is also mitogenic, anti-apoptotic, and insulinotropic for β-cells, but islet endothelial cell production is reduced in IUGR fetuses [64]. An effect that is proposed to be influenced by lower islet vascular endothelial growth factor A expression in IUGR islets [64]. In the IUGR fetal pancreas, reduced expression of growth factors or greater expression of IGFBPs could explain declines in endocrine cell mass and appear to be dependent on hypoglycemic or hypoxemic conditions associated with placental insufficiency.

Interestingly, preservation of α-cell mass appears to be dependent on an intact adrenal medulla in both IUGR and control fetuses, which has not been shown previously. In intact IUGR fetuses plasma glucagon concentrations were elevated, which has been demonstrated with an epinephrine infusion in near-term fetal sheep [65,66]. It is postulated that α-cells, via glucagon, influence β-cell function and islet formation, but the mechanisms are undefined [67,68]. In fetal mouse islets, mutation of the glucagon receptor delayed β-cell differentiation and impacted the proportion of β- to α-cells, which reduced α, β-, and δ-cell mass in adulthood [67]. Because glucagon signaling activates transcription and translation of a number of genes, including those related to adrenergic signaling and cell proliferation, the lack of adrenergic receptor stimulation may indirectly impair pancreatic endocrine mass [68]. For example, glucagon receptor deficient mice have decreased IGF, which associates glucagon signaling and the IGF axis [63,69,70]. Future work is required to understand the interaction between adrenal function and α-cell development.

## 5. Conclusions

Currently, no effective intervention to ameliorate the detrimental effects of IUGR exists. Recent reports have identified several potentially promising therapeutic strategies, including preservation of endocrine cell mass and function *in utero* [71,72]. Given the well-documented, profound effects of catecholamines on fetal development, we postulate that targeted manipulation of the adrenergic regulation of fetal growth will be a key component of a successful therapeutic approach. Endocrine cell mass appears to be regulated by other elements governing IUGR and may require supplementation of oxygen, nutrients, like amino acids, or growth factors. The results of the current study elucidate the role of the fetal adrenal medulla and induction of chronically elevated NE on fetal growth and endocrine cell composition in the context of IUGR. Further work to elucidate the underlying mechanisms and identify targets for intervention is ongoing.
